# Use of Dental Practices for the Identification of Adults With Undiagnosed Type 2 Diabetes Mellitus or Nondiabetic Hyperglycemia: Protocol for a Systematic Review

**DOI:** 10.2196/11843

**Published:** 2018-11-19

**Authors:** Zehra Yonel, Praveen Sharma, Laura J Gray

**Affiliations:** 1 School of Dentistry University of Birmingham Birmingham United Kingdom; 2 Department of Health Sciences, University of Leicester Leicester United Kingdom

**Keywords:** adults, case-finding, dental, diabetes, nondiabetic hyperglycemia, risk assessment

## Abstract

**Background:**

Type 2 diabetes mellitus (T2DM) is a growing global health burden and is expected to affect more than 590 million people by the year 2035. Evidence exists to demonstrate that dental settings have been used for risk assessment and identification of individuals who may be at high risk for T2DM or who may already unknowingly have the condition.

**Objective:**

This protocol aims to outline the methodology that will be undertaken to synthesize the literature relating to the use of primary care (nonhospital-based) dental services for the identification of undiagnosed T2DM or prediabetes—often termed nondiabetic hyperglycemia—in adult patients.

**Methods:**

This paper outlines the protocol that will be followed to conduct a systematic review and meta-analysis of the available literature. The protocol outlines the aims, objectives, search strategy, data extraction and data management methods, as well as the statistical analysis plan. The Preferred Reporting Items for Systematic Review and Meta-Analysis Protocols guidelines were followed in developing the protocol as were elements of the Cochrane handbook.

**Results:**

We expect the systematic review to be completed within 18 months of publication of this protocol and expect to see a high degree of heterogeneity in the existing literature.

**Conclusions:**

This review is of importance as it will synthesize the existing evidence base and inform future studies in the field. Following the publication of the protocol, the review will be registered on Prospective Register of Systematic Reviews. Following the completion of the review, results will be published in a suitable peer-reviewed journal.

**International Registered Report Identifier (IRRID):**

PRR1-10.2196/11843

## Introduction

Type 2 diabetes mellitus (T2DM) is a growing public health concern, accounting for 10% of the UK National Health Service (NHS) budget, a proportion predicted to rise to 17% by 2035 [1]. In addition to the 3.8 million people currently diagnosed with T2DM in the United Kingdom, it is estimated that almost 1 million UK residents have undiagnosed T2DM [2] and a further 12 million are at high risk for developing the condition [3]. Globally, the incidence of T2DM is expected to exceed 592 million by the year 2035 [4]. Individuals may remain undiagnosed for many years due to the condition being symptom-free in its early stages [5]. This has implications for the secondary prevention and management of the condition.

The UK National Screening Committee states that there are benefits to early identification of individuals at risk for developing diabetes and those with nondiabetic hyperglycemia (NDH), also known as prediabetes, as well as those with undiagnosed diabetes [3]. Advances in diabetes care mean that earlier detection may reduce the risk of complications, such as heart attacks, stroke, and blindness [6,7]. Evidence exists that diabetes is preventable in those at high risk [8]. Hence, the NHS has developed the Diabetes Prevention Programme. Novel approaches to identify cases of previously undiagnosed diabetes and high-risk individuals may result in improved health outcomes, improved quality of life for patients, and reductions in cost to the NHS.

In the United Kingdom, 60% of the adult population routinely attends high-street dentists for regular check-ups, even when they have no concerns [9]. Furthermore, patients’ diabetes status influences their dental management; therefore, it is useful for dentists to be aware of this condition. Using dental visits for early diabetes detection represents a unique opportunity to access large proportions of the population for diabetes screening.

The National Institute for Care and Health Excellence pathways exist for allied health care professionals, including dentists, relating to risk assessment for diabetes [10] in community and primary care settings. Some UK community pharmacists perform risk assessment of patients for diabetes. However, using primary dental practices has not been widely explored as an option for identifying high-risk individuals and, therefore, represents a potential missed opportunity.

Studies conducted in the United States have indicated that dental practices can be effective in identifying those at high risk for diabetes [11-13]. There have also been studies in Europe that support these findings [14-16]. Dental practices in the United Kingdom may also offer the opportunity for proactive, early case detection of high-risk individuals and those who already unknowingly have T2DM.

Despite the existing literature published in the field to date, no published systematic reviews have synthesized the current evidence base for the use of primary care dental settings for the detection of T2DM and NDH. The aim of this protocol is to outline the design of a systematic review investigating the available literature for utilizing dental settings to case-find previously undiagnosed T2DM and NDH. The primary aim of the review will be to establish the identification rate of previously undiagnosed diabetes and NDH and the opinions, benefits, and barriers related to case-finding T2DM and NDH in dental settings.

## Methods

### Protocol Guidelines Followed

The intention to conduct a systematic review is evidenced through registration with the prospective register of systematic reviews (PROSPERO), the Web-based international prospective register of systematic reviews, at the time of protocol conception. This protocol followed the Preferred Reporting Items for Systematic Review and Meta-Analysis Protocols 2015 statement [17] alongside elements from the Cochrane handbook for systematic reviews [18].

### Review Question and Objective

This review aims to identify the literature relating to the use of primary care (nonhospital-based) dental services for the identification of undiagnosed T2DM and NDH—often termed prediabetes—in adult patients. The review will have a particular focus on the pick-up rate of new cases of NDH and T2DM with the following additional questions, which this review will aim to answer:

- What methodology was utilized within the dental practice for case-finding?
- What were the recruitment rates within the studies?
- What are the opinions of patients and health care professionals relating to such services?
- What are the reported barriers to uptake of any such implemented services?
- What are the reported benefits of utilizing such services?

The Population Intervention Control Outcome format was followed; this format involves clearly identifying participants, intervention, comparator, and outcome within the research question. For this review, these were patients (P) aged >18 years attending primary care (nonspecialist practice) dental services. The specific intervention (I) for this review is focused on the risk assessment methods used for identification of T2DM or NDH (prediabetes). It is recognized that a number of methods for risk assessment of patients have been discussed in the literature. This includes using questionnaires, finger-prick point-of-care testing, gingival crevicular fluid samples, and both one- and two-stage procedures utilizing a combination of these methods; these differing methods will act as the comparators (C). This review will attempt to capture and compare the full range of assessments used.

### Outcome Measure

The primary outcome measure for this systematic review is the identification of patients with NDH or T2DM using risk assessment methods in dental care settings. The secondary outcomes include identification of methodologies utilized in the dental practice for case-finding, establishing recruitment rates in the studies, and gaining insight into the opinions of patients and health care professionals relating to case-finding. In addition, the review will aim to enhance the understanding of reported barriers to uptake of any such implemented services and any reported identified benefits to utilizing dental settings to case-find NDH and T2DM.

### Inclusion and Exclusion Criteria

The inclusion and exclusion criteria have been presented in Textboxes 1 and 2, respectively.

**Inclusion criteria.**

- Adults aged >18 years
- English language literature
- Diabetes risk assessment conducted
- Risk assessment based in primary care dental settings

**Exclusion criteria.**

- Non-English language
- Animal studies
- Nonprimary care dental settings

**Table 1:** Draft of search strategy to be used.

Query	Items found
Search (((((((((screening) OR “risk assessment”) OR “case detection”) OR “case finding”) OR “identification”) OR “risk detection”) OR “diagnosis”)) AND ((((((((((“diabetes mellitus”) OR “diabetes”) OR “type 2 diabetes”) OR “type two diabetes”) OR TTDM) OR T2DM) OR prediabetes) OR Pre-diabetes) OR “non diabetic hyperglycaemia”) OR NDH)) AND (((((dental) OR dentistry) OR “primary dental care”) OR “general dental practice”) OR dentist)	1466
Search ((((dental) OR dentistry) OR “primary dental care”) OR “general dental practice”) OR dentist	73,7631
Search (((((((((“diabetes mellitus”) OR “diabetes”) OR “type 2 diabetes”) OR “type two diabetes”) OR TTDM) OR T2DM) OR prediabetes) OR Pre-diabetes) OR “non diabetic hyperglycaemia”) OR NDH	600,088
Search ((((((screening) OR “risk assessment”) OR “case detection”) OR “case finding”) OR “identification”) OR “risk detection”) OR “diagnosis”	3,232,401

### Search Strategy

To identify the eligible literature, the following electronic bibliographic databases will be searched: Medical Literature Analysis and Retrieval System Online, PubMed, The Cochrane Library, and Web of Science. The reference lists of all eligible full texts will be searched for additional papers for inclusion. In addition to electronic databases, trial registries such as Clinicaltrials.gov will be searched.

The search strategy will include terms relating to or describing the identification of NDH and T2DM in dental settings. The search terms will be adapted for use with other bibliographic databases in combination with database-specific filters for controlled trials, where these are available (Table 1). There will be restrictions to English language only. Searches will be limited to 1950—search date to allow for replication. Furthermore, the searches will be rerun just before the final analyses and further studies retrieved for inclusion.

### Risk of Bias

This review will not be restricted to only randomized controlled trials. A published and validated risk of bias assessment tool appropriate to the study type will be utilized [19] independently by two reviewers to determine the bias associated with included papers. The tool will be specific to the study design, and all papers included in the review will be appraised by the authors. Disagreement will be resolved by discussion, and where required, a third author will be consulted.

### Data Extraction and Data Management

The search will be undertaken; all returned papers will have title and abstract screened independently by two researchers to establish studies that potentially meet the inclusion criteria. Calibration exercises will be undertaken until authors are consistent in their acceptance of suitable papers. Where there is disagreement regarding a paper’s exclusion, consensus will be reached by a third reviewer. For the papers included, full text will be reviewed by the two authors, and any further exclusions will be determined by consensus and agreement among authors with reason for exclusions reported. Reason for exclusion at full-text stage will be recorded.

Electronic data extraction forms will be developed and piloted. The standardized prepiloted form will be used to extract data from included studies to assess the study quality and evidence synthesis. Extracted information will include the following: study setting, population and participant demographics and baseline characteristics; details of the intervention and control conditions; study methodology; recruitment, completion, and pick-up rates; outcomes and times of measurement; indicators of acceptability to users; suggested mechanisms of intervention action; and information about assessment of the risk of bias. This information will be collected independently by the two reviewers with discrepancies identified and resolved through discussion and, if required, with the third author. Where data are missing, attempts will be made to retrieve the data by contacting study authors. The key data to be extracted are presented in Textbox 3.

**Key data to be extracted.**

- Study ID:
- Reviewer ID and name:
- Date of completion of this form:
- Title of report:
- Source [journal year; volume: pages]:
- Authors:
- Type of report [eg, full paper or abstract or unpublished]:
- Country where the trial was conducted:
- Funders of the study:
- Dates study was conducted:
- Type of study design [eg, observational or clinical trial (randomized, parallel, or cluster, etc)]
- Was the study multicenter? If so, how many centers were there?
- Risk of bias criteria—[dependent on study type]
- Inclusion criteria
- Exclusion criteria
- Participant information
  - Age
  - Gender
  - Ethnicity
- Risk assessment method used
- Screening process
- Recruitment rates
- Prevalence of undiagnosed type 2 diabetes mellitus (T2DM) and nondiabetic hyperglycemia (NDH)
- Method for diagnosis of T2DM or NDH
- Stakeholder opinions [patients or dental team or health care professionals, etc]
- Barriers to risk assessment in dental settings
- Key findings
- Additional comments

Electronic data extraction form will be developed in Microsoft Excel with care to ensure that updated versions do not overwrite previous iterations of extracted data.

### Strategy for Synthesis

If the included studies are sufficiently homogenous, a quantitative synthesis will be undertaken. However, it is anticipated that the included studies will demonstrate high levels of heterogeneity, resulting in a descriptive synthesis approach. The descriptive synthesis will be structured around the primary and secondary outcomes of the review. It is anticipated that there will be limited scope for meta-analysis because of the range of different outcomes measured, although we expect the percentage of cases of undiagnosed T2DM and NDH to be well reported across the assumed small number of existing studies. However, where studies have used the same risk assessment strategy with the same outcome measure, results will be pooled and meta-analysis undertaken. Any meta-analysis conducted will use a random effects model to pool data given the expected high levels of heterogeneity expected between studies.

## Results

We expect the systematic review to be completed within 18 months of the publication of this protocol and expect to observe a high degree of heterogeneity in the existing literature.

## Discussion

This review is of importance as it will synthesize the existing evidence base and inform future studies in the field. Following the publication of the protocol, the review will be registered on PROSPERO. Following the completion of the review, results will be published in a suitable peer-reviewed journal.

## Abbreviations

NDH: nondiabetic hyperglycemia
NHS: National Health Service
PROSPERO: prospective register of systematic reviews
T2DM: type 2 diabetes mellitus
